# The development and evaluation of a self-marking unit to estimate malaria vector survival and dispersal distance

**DOI:** 10.1186/s12936-019-3077-3

**Published:** 2019-12-23

**Authors:** Adam Saddler, Katharina S. Kreppel, Nakul Chitnis, Thomas A. Smith, Adrian Denz, Jason D. Moore, Mgeni M. Tambwe, Sarah J. Moore

**Affiliations:** 1 Ifakara Health Institute, Environmental Health and Ecological Sciences, P.O. Box 74, Bagamoyo, Tanzania; 2 Swiss Tropical & Public Health Institute, Socinstrasse 57, 4051 Basel, Switzerland; 3 University of Basel, Petersplatz 1, 4001 Basel, Switzerland; 4 Nelson Mandela African Institution of Science and Technology, P.O. Box 447, Tengeru, Tanzania

**Keywords:** Mark-release-recapture, Anopheles, Mosquito, Vector, MMRR, Release-recapture, Dispersal, Survival

## Abstract

**Background:**

A clear understanding of mosquito biology is fundamental to the control efforts of mosquito-borne diseases such as malaria. Mosquito mark-release-recapture (MMRR) experiments are a popular method of measuring the survival and dispersal of disease vectors; however, examples with African malaria vectors are limited. Ethical and technical difficulties involved in carrying out MMRR studies may have held back research in this area and, therefore, a device that marks mosquitoes as they emerge from breeding sites was developed and evaluated to overcome the problems of MMRR.

**Methods:**

A modified self-marking unit that marks mosquitoes with fluorescent pigment as they emerge from their breeding site was developed based on a previous design for *Culex* mosquitoes. The self-marking unit was first evaluated under semi-field conditions with laboratory-reared *Anopheles arabiensis* to determine the marking success and impact on mosquito survival. Subsequently, a field evaluation of MMRR was conducted in Yombo village, Tanzania, to examine the feasibility of the system.

**Results:**

During the semi-field evaluation the self-marking units successfully marked 86% of emerging mosquitoes and there was no effect of fluorescent marker on mosquito survival. The unit successfully marked wild male and female *Anopheles gambiae* sensu lato (s.l.) in sufficiently large numbers to justify its use in MMRR studies. The estimated daily survival probability of *An. gambiae* s.l. was 0.87 (95% CI 0.69–1.10) and mean dispersal distance was 579 m (95% CI 521–636 m).

**Conclusions:**

This study demonstrates the successful use of a self-marking device in an MMRR study with African malaria vectors. This method may be useful in investigating population structure and dispersal of mosquitoes for deployment and evaluation of future vector control tools, such as gene drive, and to better parameterize mathematical models.

## Background

A clear understanding of mosquito biology is fundamental to the effective control of mosquito-borne diseases including malaria, dengue, Zika and lymphatic filariasis. While novel vector control tools such as spatial repellents [1, 2], attractive targeted sugar baits (ATSBs) [3, 4] and gene-drive systems [5], continue to be developed, our understanding of several key aspects of mosquito biology remains limited. Therefore, predicting how these novel tools will function in real world settings is difficult. Planning and evaluation of these new tools, in particular gene drives, require a detailed understanding of mosquito dispersal and survival of both male and female mosquitoes [6].

Mosquito mark-release-recapture (MMRR) studies have been one of the most widely used ways to obtain field estimates of daily mosquito survival, population size, duration of the gonotrophic cycle and dispersal distances [7, 8], since tracking individual mosquitoes over distances larger than a few metres remains infeasible. A review in 2014 of MMRR studies with female mosquitoes identified 774 separate MMRR experiments covering 58 mosquito species that are of importance for human disease transmission [8]. However, there is a paucity of studies on African malaria vectors with only 11 studies on *Anopheles gambiae* sensu lato (s.l.) identified. Daily survival is the most studied parameter in MMRR studies on malaria vectors [9–12], although population estimates [6, 11, 13, 14] and behavioural studies [15–17] are also conducted. Detailed analysis of dispersal distances were carried out by Gillies with *An. gambiae* in Tanzania [9] and Costantini et al. [18] with *An. gambiae* s.l. in Sudan. Considering the burden of malaria across Africa and the variety of ecological settings across the continent, there are surprisingly few field-estimates of the essential entomological parameters for optimizing the implementation of focused vector control [19] and the design of cluster randomized field trials [20].

The numerous ethical and technical difficulties involved in carrying out MMRR studies have been described in detail [7, 21] and guidance on the safety of MMRR studies is available [22]. The methods used in each step of an MMRR study, from sourcing, marking, releasing, recapturing and handling mosquitoes, has the potential to disrupt the normal behaviour and survival of the wild mosquitoes. Visual markers such as fluorescent powders and paints have been the main method to mark anopheline mosquitoes [8, 21], but reports on their impact on mosquito survival are inconclusive [21, 23–25]. This variation in results may be caused by species or environmental differences but is most likely due to the different application methods, the degree of mosquito handling, the amount and brand of pigment applied [25]. It is therefore important to measure the impact of the marking method on survival when using a new marker and species combination.

At the forefront of ethical concerns is the risk of releasing highly competent, potentially disease transmitting, mosquitoes and so careful consideration is needed for a study to be carried out safely [22]. It could be argued that as MMRR studies capture far more mosquitoes than they release they mitigate any risk of increasing the local vector population in the short-term [22]. However, MMRR studies that release laboratory strains of mosquitoes have potential longer-term risks. Laboratory strains are often selected for increased longevity, strong human feeding preference or high susceptibility to parasite infection. All of these characteristics could dramatically increase vector competence if expressed in wild vector populations through inter-breeding with released individuals. In addition to the ethical concerns, using laboratory reared mosquitoes with different survival rates and dispersal behaviours to their wild counterparts will misrepresent the very parameters that are being estimated [26].

Sourcing mosquitoes from wild populations is preferable, and is in fact the most common method used in MMRR studies with *Anopheles* [8], but it has its own limitations. Collection of large numbers of mosquitoes is required for MMRR studies, but the capturing and handling of adult mosquitoes before marking may cause stress and consequently impact on their survival and dispersal. Furthermore, information on mosquito age or infection status is unknown at the time of marking and may influence results.

In order to overcome these disadvantages a self-marking unit first developed by Niebylski and Meek to mark *Culex quinquefasciatus* [27] as they emerge from the breeding sites was adapted and optimized for use with *Anopheles arabiensis*, *Anopheles funestus*, and *Anopheles gambiae* sensu stricto (s.s.).

## Methods

### Study site

**Fig. 1:**
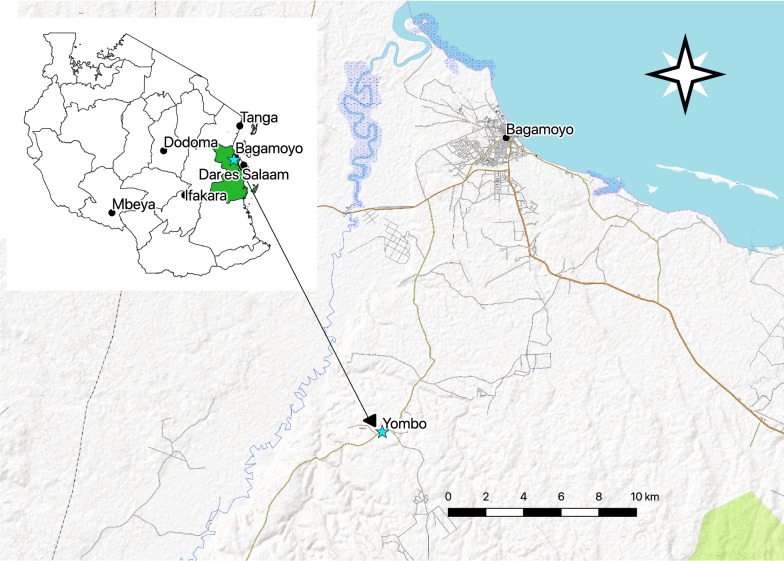
Map highlighting Yombo village (6° 35′ 01.0″ S, 38° 50′ 48.4″ E), the site for the MMRR study. Yombo is approximately 17 km south of Bagamoyo town and 5 km east of the Ruvu river in the Pwani region (in green) of Tanzania. The Bagamoyo branch of Ifakara Health Institute, where the semi-field work was conducted, is based to the west of Bagamoyo town centre. Base maps were provided by Open Street Map Contributors [50] through the QGIS plugin [33]. Map data copyrighted by OpenStreetMap contributors and available from https://www.openstreetmap.org

The marking unit was first evaluated in the Ifakara Tunnel, a large screened cage at the Bagamoyo branch of the Ifakara Health Institute (IHI), Tanzania [28]. The tunnel provides ambient environmental conditions where experiments can be conducted safely with disease-free laboratory-reared mosquitoes. Field evaluations were conducted at Yombo village, Tanzania (6° 35′ 01.0″ S, 38° 50′ 48.4″ E, Fig. 1) located approximately 17 km south of Bagamoyo town and 5 km east of the Ruvu river in the Pwani region of Tanzania. Bagamoyo district experiences an annual rainfall of 800–1000 mm and an average temperature of 28 °C. Two rainy seasons replenish permanent breeding sites such as streams and ponds and create temporary breeding sites such as puddles. Malaria is endemic in Bagamoyo and the main vectors are *Anopheles arabiensis*, *An. funestus*, and *An. gambiae* s.s. [29].

### Experimental design

Experiments to optimize the self-marking unit were first conducted under semi-field conditions to determine (i) the efficiency of the self-marking unit measured as the percentage of marked mosquitoes; (ii) if marking had an impact on survival; and (iii) if pigment transfer to unmarked mosquitoes occurred during mosquito collection. Experiments were conducted using *An. arabiensis* (Kingani strain) reared under standard laboratory conditions previously described [30].

This was followed by preliminary field experiments in breeding sites located close to the IHI, Bagamoyo Branch to measure the number of mosquitoes marked by the self-marking units. Marking units covering natural breeding sites were compared to units which were placed next to the breeding site and had pupae placed in bowls underneath them from the surrounding breeding site. Finally, to test the feasibility of the self-marking unit and possible applications in studies with wild malaria vectors, a small MMRR field trial was conducted in Yombo Village.

### Self-marking unit design

**Fig. 2:**
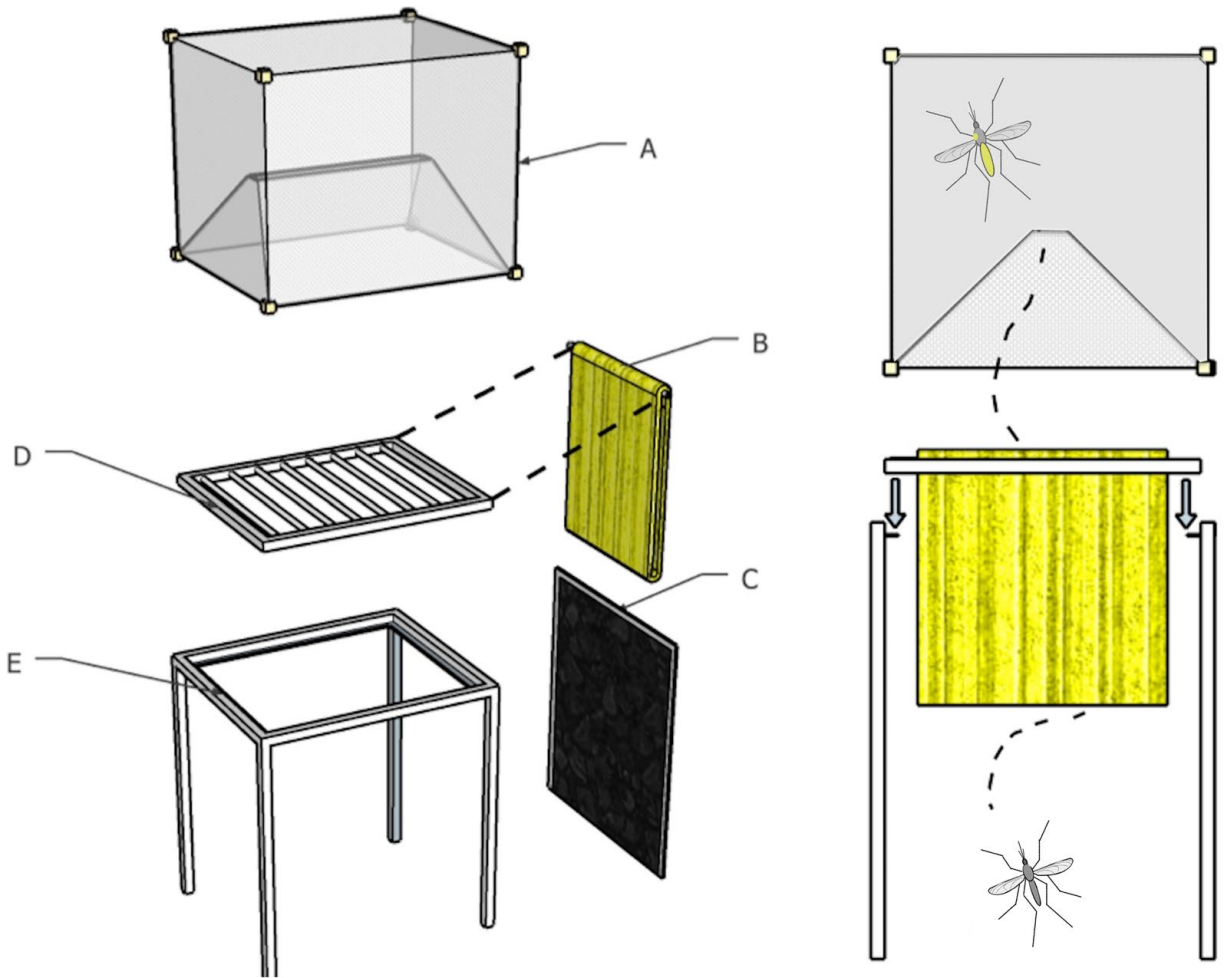
The self-marking unit adapted from Niebylski and Meek [27]. Left panel: 3D model of the marking unit indicating the key components. A—an exit trap used previously for hut trials as a window trap [31]. A slit in the netting allows mosquitoes to pass through in one direction thus collecting the mosquitoes after they have passed through the marking unit. The exit traps were only used when marked mosquitoes need to be retained as in the semi-field experiments, B—cloth impregnated with fluorescent pigment, C—black cloth side panels attached to the frame with Velcro, D—detachable marking grid from which the impregnated cloth hangs. It can be removed without tools and replaced with another grid containing a new colour, E—frame to hold the marking grid made from 2 × 2 cm^2^ metal tubing. Right panel: side view of the unit to show the frames’ internal lip on which the marking grid sits. The path of an emerging mosquito is shown passing through the marking grid and picking up fluorescent pigments

The core component of the self-marking unit is the marking grid containing cloth impregnated with fluorescent pigment (Fig. 2). As adult mosquitoes emerge from pupae and they must pass through the layers of impregnated cloth to take their first flight and as a result are marked with the pigment. To allow daily colour change the unit was designed so that the marking grid can easily be removed and replaced with a new grid containing a different colour of fluorescent. Five colours from the A series range of fluorescent pigments were selected for the study: Laser Red 3, Flame Orange 4, Solar Yellow 7, Stellar Green 8 and Comet Blue 80 (SWADA, Cheshire, UK). For the remainder of the manuscript the colours will be referred to as pink, orange, yellow, green and blue, respectively. White loose weave cotton cloth was purchased from a local fabric store and cut into 50 × 50 cm^2^. The cloth was placed in a large plastic bag with half a cup of fluorescent pigment (approx. 125 g) and shaken until an even coating of colour was achieved.

### Marking grid dimensions

The marking grid was made from 2 × 2 cm wide, square metal tubing and measured 54.5 × 45 cm with interspersed metal rods at 5 cm intervals spanning the 45 cm width. Impregnated cloth was attached to the grid by looping a 50 × 50 cm piece of cloth around one rod and by stapling the ends of the cloth together at the bottom—this was done for all eight rods of the marking grid. The frame to hold the marking grid was also made from 2 × 2 cm^2^ metal tubing and measured 59 × 49.5 cm with 62 cm long legs. An inner lip of metal sheeting measuring 2 cm (Fig. 2) allowed the marking exit grid to sit snugly within the frame without the use of tools for attachment.

**Fig. 3:**
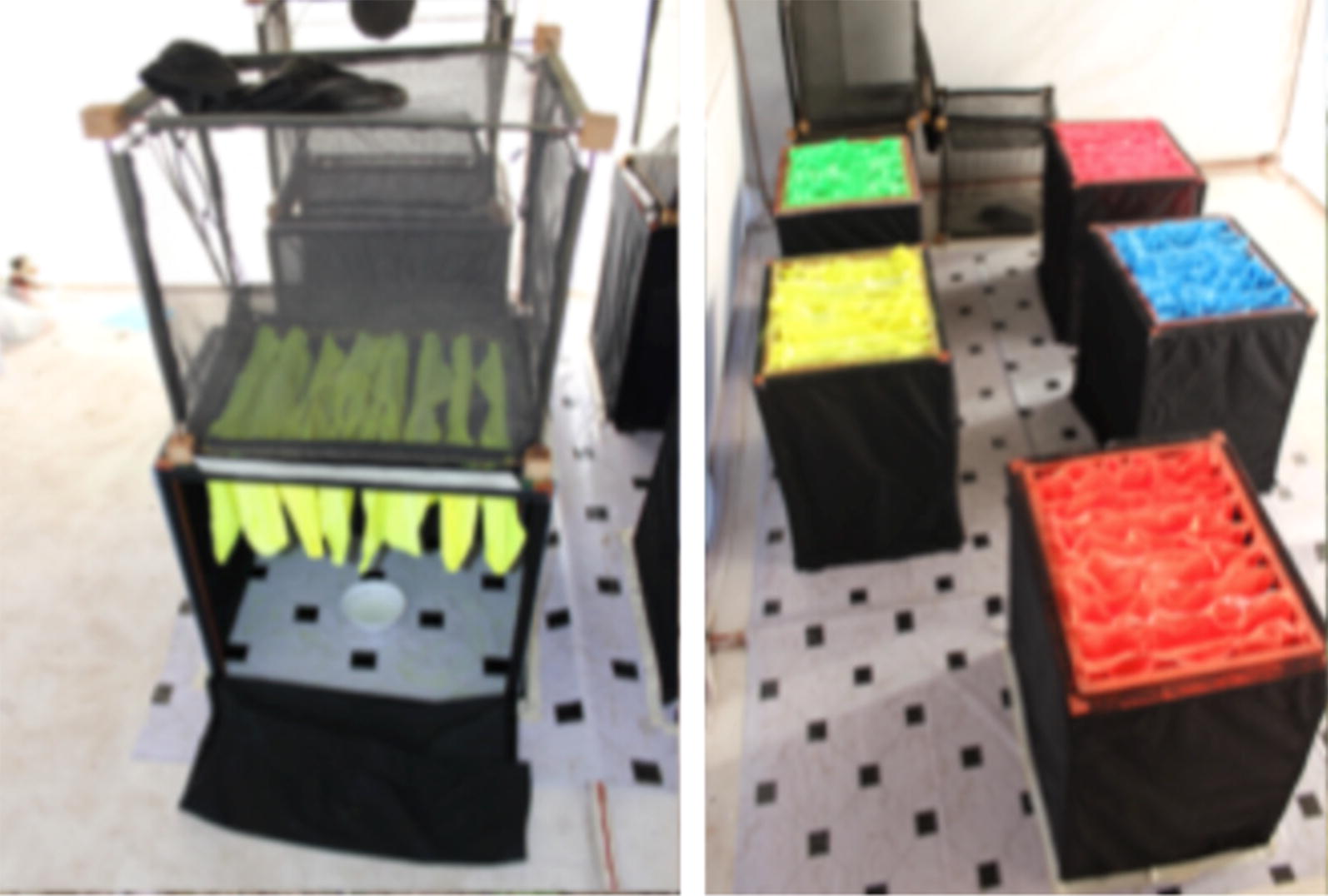
Evaluation of the self-marking units under semi-field conditions. Left panel: an open side panel showing a bowl of *An. arabiensis* pupae underneath. The side panel is closed for the experiment and emerging adult mosquitoes fly and bump their way through the layers of cloth to exit the marking grid and into the exit trap. Adults are collected from the exit trap by aspiration through the cloth sleeve on top of the trap. Right panel: the units set-up as intended for field use with side panels closed and exit traps removed. Five units each containing a different colour marker used in the study

Black cloth was attached with Velcro to all sides of the frame to enclose the unit and to ensure that the only exit was up through the impregnated cloth. Preliminary experiments indicated that dark cloth that fitted tightly around the frame increased exiting rates of laboratory reared *An. arabiensis* from the units compared to netting or a loose-fitting funnel shape. The dimensions of the marking units allowed the attachment of exit traps previously designed for trapping mosquitoes exiting windows in hut studies [31] (Figs. 2, 3). The exit traps were used in both semi-field and field experiments to capture mosquitoes exiting the marking units and thus enabling mosquito collection and the examination of pigment transfer to the mosquitoes.

### Marking success and survival of laboratory reared *Anopheles arabiensis*

Six self-marking units with exit traps attached were placed in a large experimental chamber (5 m × 3 m × 2.1 m) under semi-field conditions. Each unit contained a different colour of fluorescent marker (orange, blue, yellow, green, pink) or a pigment-free cloth as a control (Fig. 3). At 17:00 h East African Time (EAT), 60 laboratory reared *An. arabiensis* pupae, in a bowl with water, were placed under the self-marking unit and left overnight to emerge. At 10:00 h EAT the following day, mosquitoes that had emerged and had been captured in the exit traps were transferred to holding cups and provided with cotton wool soaked in 10% sucrose solution. Any pupae that had not emerged and remained in the bowl under the marking unit were left for a further 24 h for a second collection from the exit traps. The marking process was repeated four times over 8 days. Holding cups were labeled with a unique individual identification number that identified the exit trap, colour pigment and date of emergence. Each holding cup contained a maximum of twenty mosquitoes and was immediately transferred to a screened laboratory and held at ambient temperature and humidity with access to sugar. Survival of the mosquitoes was recorded daily until all mosquitoes were dead. Each day all dead mosquitoes were removed and checked for florescent powder using a UV-torch and microscope.

### Pigment transfer during recapture of laboratory *Anopheles arabiensis* using CDC light traps or aspiration

The self-marking device relies on mosquitoes picking up fluorescent pigment when they contact impregnated cloth. It is therefore not unreasonable to assume that mosquitoes could also pick up the pigment when they contact mosquitoes that already have been marked especially when forced into the confined space of traps and collection tools. Pigment transfer during collection was assessed for three common methods of mosquito sampling: the Centers for Disease Control and Prevention light trap (CDC-LT), the battery powered Prokopack aspirator [32] and standard mouth aspirator.

Five CDC-LTs were hung individually in five large cages (120 × 120 × 120 cm). At 18:00 h EAT, 20 mosquitoes were introduced to each cage: 10 mosquitoes that were marked using the self-marking unit and 10 unmarked mosquitoes. The traps were left to run overnight and the number of mosquitoes with colour pigment was assessed the following day. If there were more than 10 marked mosquitoes then it was deemed pigment transfer had taken place. Due to the size of the cages, the CDC-LTs did not use an odour-lure as the phototactic response was sufficient to attract the majority of released mosquitoes to the trap. However, any mosquitoes not in the trap and still in the cage were adjusted for when recording results. Five replicates of each of the five colors were conducted over five nights.

A similar method was used to examine pigment transfer while using a Prokopack or manual aspiration. Five marked and five unmarked mosquitoes were released into each of the large cages. Collections were conducted 10 min after the mosquitoes were released. Two manual aspiration methods were examined; (i) aspiration of mosquitoes individually but transferred to the same cup (ii) group aspiration (3–5 mosquitoes at a time) before transferring to the same cup. Again, five replicates were conducted for each colour pigment. All replicates were completed in a single day after which they were transferred to the laboratory for counting (1–2 h after collection).

### Field testing

#### Preliminary trials in natural breeding sites and the development of a pupae collection method

The original self-marking device for *Culex quinquefasciatus* mosquitoes was designed to be used over natural breeding sites [27]. As the breeding sites of mosquito species can vary significantly, several different prototypes were developed to cover the breeding sites of *Anopheles arabiensis*, *An. funestus*, and *An. gambiae* s.s. The basic marking unit covers a breeding site of 55 cm × 45 cm and therefore it can fit over small temporary breeding sites favoured by *An. gambiae*, such as water-filled hoof prints or puddles. The Velcro side panels were removed and prototypes with tarpaulin skirt extensions were made to cover ditches and larger breeding sites. A floating unit was designed in order to mark *An. arabiensis,* which is often found in rice paddies, and *An. funestus*, often located in more permanent water bodies like swamps and ponds. A method to increase the mosquito numbers passing through the marking unit was also tested by collecting pupae and stage four larvae from the surrounding breeding site and placing them in a bowl under the unit.

**Fig. 4:**
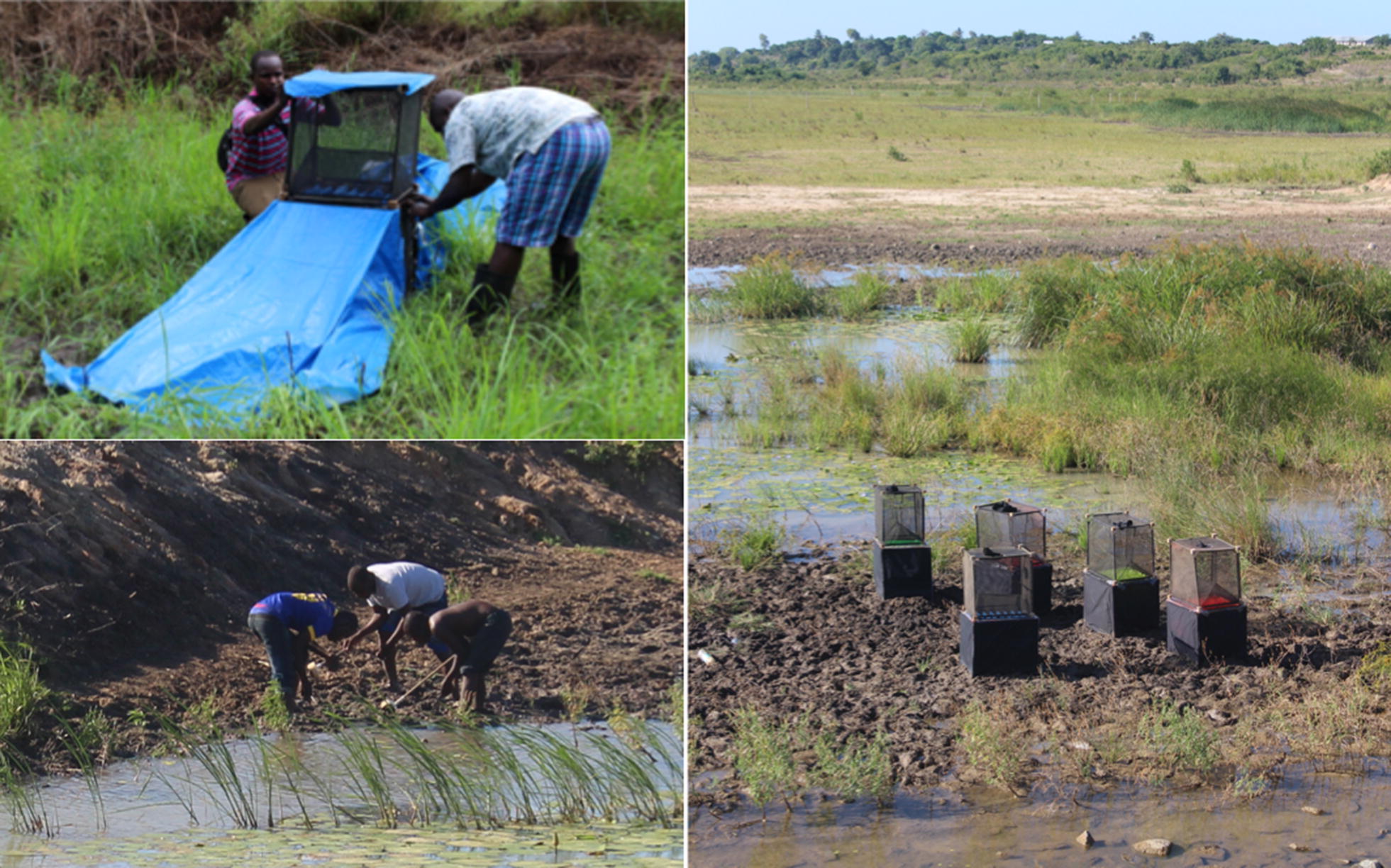
Self-marking units in the field with exit traps to measure exiting rates of mosquitoes from natural breeding sites and sites were the numbers of pupae were manipulated. Top left: self-marking UNIT with tarpaulin skirt extension to cover a ditch where *Anopheles* larvae were found. Right: five basic self-marking units containing the five pigments used in the study (pink, orange, yellow, green and blue). Bottom left: pupae collection with dippers. Larval dippers were first used to identify productive breeding sites and then to collect pupae to be placed under the self-marking devices

Five of the basic marking units were deployed for 10 days over a breeding site close to IHI Bagamoyo Branch and trapped emerging mosquitoes in attached exit traps (Fig. 4). For five of these days, the units were deployed on natural breeding sites and for the other 5 days, the devices were deployed in the same area but contained small bowls underneath, where pupae and stage four larvae were placed after collection by the field team.

### MMRR field trial

#### Estimation of released mosquitoes

A productive breeding site for *Anopheles* mosquitoes was identified in Yombo and one marking unit was placed adjacent to the breeding site for 5 days. On each day, a marking grid with a new colour was introduced. Trained technicians collected pupae from an area within 20 m of the marking unit for approximately 1 h each day. As species identification of pupae can be difficult, breeding sites were sampled where predominantly *Anopheles* larvae were found. The collected pupae were counted and placed in a bowl under the marking system at 18:00 h EAT. The following day, the pupae remaining in the bowl were counted and subtracted from the previous day’s total to calculate the number of mosquitoes that had emerged through the marking grid.

### Recapture methods

**Fig. 5:**
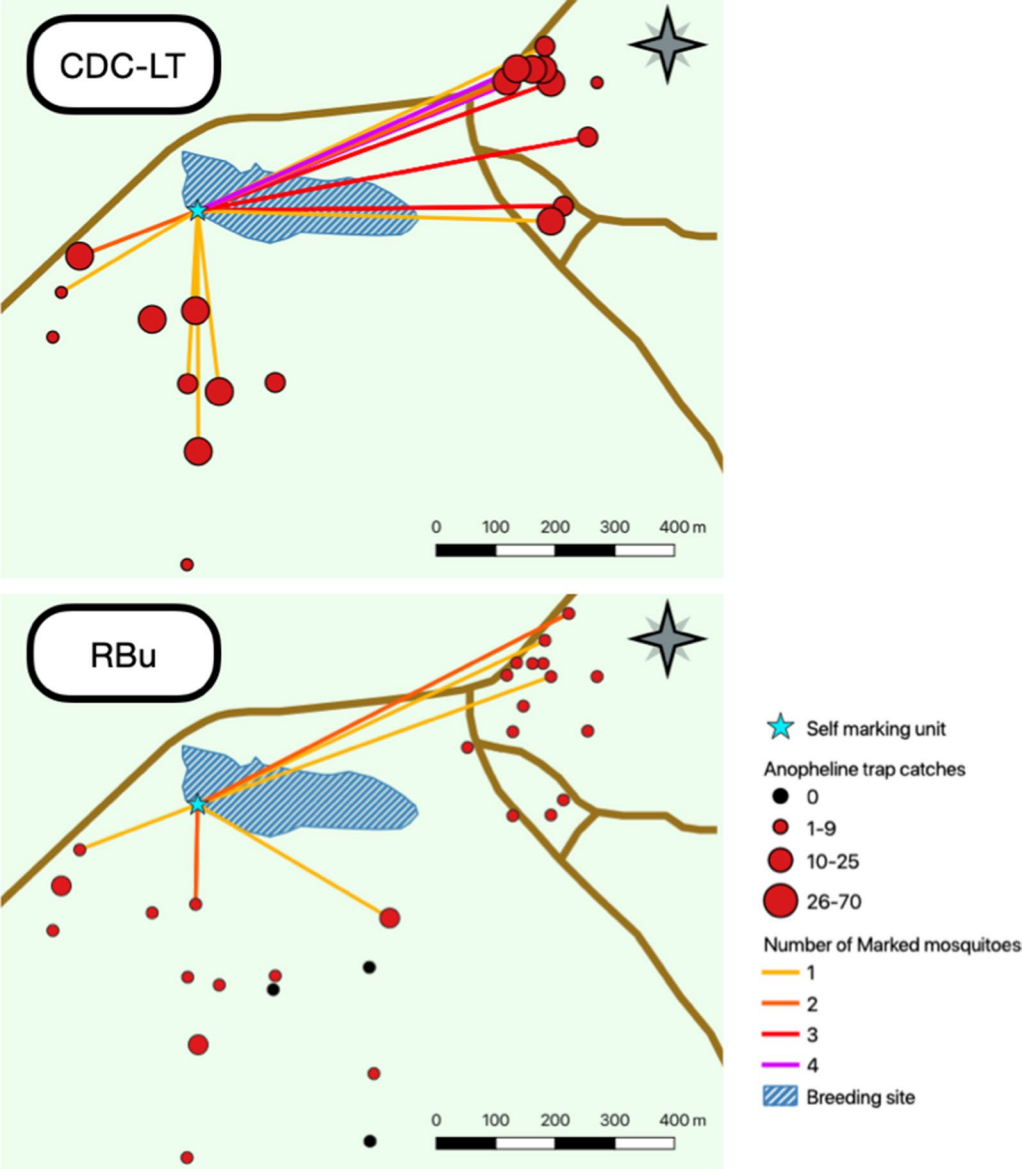
Distribution of marked and unmarked female Anopheline mosquitoes caught by CDC light traps (top) or resting bucket traps (bottom). Size and colour of circles indicate the total number of female anopheline mosquitoes (unmarked and marked) caught in each trap for the duration of the trapping (12 days). Marked and recaptured mosquitoes are indicated by lines dispersing from the self-marking unit—also indicating the total number of marked mosquitoes caught at the final trap location

Adult mosquito collections were conducted for 12 days, following the first marking day, among thirty houses upon written informed consent of the household head. GPS coordinates of each household were taken to calculate the distance and direction of the household in relation to the marking unit using QGIS software version 3.6.0 [33]. No other household information was recorded. To maximize recapture probability, recapture was focused in an area within 1 km of the breeding site (Fig. 5). Outdoor resting mosquitoes were sampled from all 30 households with resting buckets (RBu) [34] and indoor host seeking mosquitoes were sampled among 20 of the 30 households using CDC-LTs.

Ten RBu were deployed at each sampling site and were placed facing the household roughly five metres away in all directions. Mosquitoes were collected at 06:00 each morning using a Prokopack aspirator [32]. The sum of mosquitoes caught in the ten buckets was considered the RBu catch for 1 day for that household. CDC-LTs were deployed from between approximately 18:00 h and 06:00 h every night by hanging them approximately 1.5 m above ground and close to the foot of a bed in which an individual slept under an insecticide-treated bed net. CDC-LT catch bags were collected in the morning shortly after the resting buckets were sampled. Mosquito identification and inspection of each mosquito for fluorescent pigment was performed daily using UV-torch and microscope.

### Data management and statistical analysis

All data were collected first by hard copy and then transferred into Excel using double entry. Analyses were carried out with R statistical software v3.5.2 [35].

### Survival analysis of laboratory *Anopheles arabiensis*

Mosquito survival was measured in days and analysed with a mixed-effects Cox model using the “coxme” package in R [36]. Colour was included in the model as a fixed factor with 6 levels (5 colours and control). Night of emergence and mosquito sex were also included as fixed factors. Round and cup ID number were included as random factors. From the mosquitoes that passed through the coloured marking grids, only mosquitoes identified as with a colour pigment were included in the survival analysis.

### Marking success of laboratory *Anopheles arabiensis*

Marking success was determined by the percentage of mosquitoes marked after passing through the marking unit and was analyzed using binomial Generalized Linear Mixed Effects Models (GLMM). The colour of the pigment, mosquito sex and emergence day were treated as fixed factors and round of experiment was included as a random factor. An individual random effect was included in the model to account for overdispersion after it was identified in the initial models. The analysis was carried out using the “lme4” package [37]. Post hoc pairwise comparisons using Tukey contrasts were preformed between each colour pigment using the “multcomp” package [38].

### MMRR field trial

Summary statistics were used to describe the number of mosquitoes marked by the units and the total number of marked and unmarked mosquitoes captured during the trapping. Data from the mark-recaptured mosquitoes was used to calculate daily survival rates and mean distance travelled (MDT) by *An. gambiae* s.l.

The MDT was calculated using a correction factor that takes account of uneven sampling effort over distance [39, 40]. Briefly, the sampling area was divided into four concentric annuli separated by 200 m. For each annulus the number of traps and area were used to calculate a correction factor (CF),

$$AnnulusCF = \frac{Area\;of\;annulus}{Total\;trapping\;area} \times total\;number\;of\;traps.$$

The correction factor was then applied to the observed recapture numbers in order to calculate the estimated recapture (ER) per annulus,

$$ER = \frac{Number\;of\;observed\;recaptures\; in \;annulus}{Number\;of\;traps \;in \;annulus} \times AnnulusCF.$$

Finally the MDT was calculated as

$$MDT = \frac{{\sum {\left( {AnnulusER \times annulus\;distance} \right)} for\;all\;annuli}}{Total\;number\;of\;ER}.$$

The MDT was first calculated using recaptured mosquitoes from all 12 days of recapture; a “first flight” MDT was then calculated using only mosquitoes recaptured in the 3 days following marking. The calculation of MDT makes simplifications because it treats each location as equally attractive to mosquitoes, and assumes dispersal is equal in all directions.

The daily survival probability was estimated using an exponential model [9], where the log_10_ (x + 1) number of recaptures is regressed against day after marking and the antilog_10_ of the slope of the regression is the daily survival probability. Average life expectancy (ALE) was derived from the survival estimate [41].

## Results

### Survival of laboratory *Anopheles arabiensis*

**Fig. 6:**
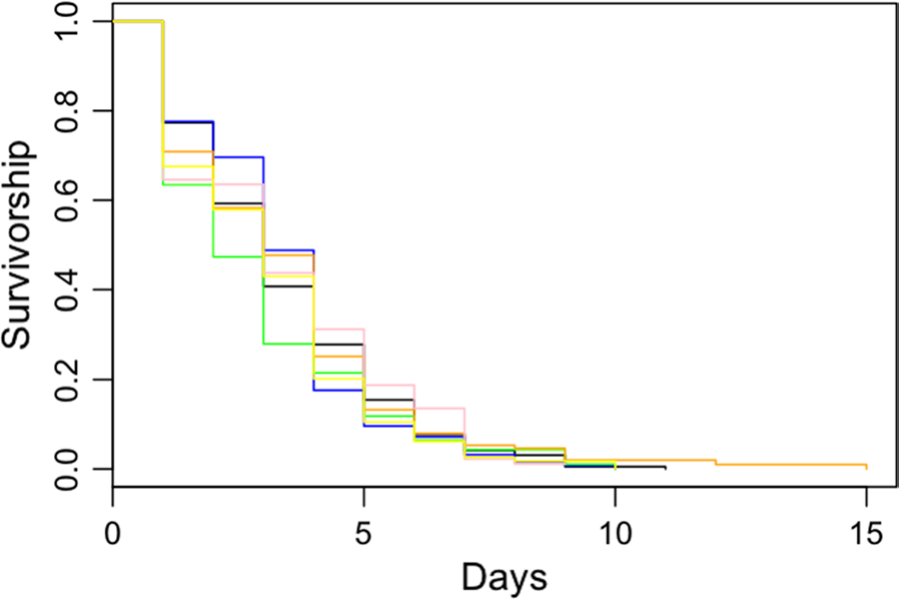
Kaplan–Meier survival curves of laboratory reared *An. arabiensis* mosquitoes marked with the self-marking unit. Five colours (blue, pink, yellow, orange and green) were examined for their impact on mosquito survival. The colours of the lines represent mosquitoes marked with that colour. The black survival curve is from unmarked controls. There was no significant impact of individual colours or marking as a whole

The daily survival of marked mosquitoes in the laboratory was not significantly different from unmarked mosquitoes independent of the pigment colour; Blue (HR = 1.12, 95% CI 0.81–1.43, p = 0.48), Green (HR = 1.34, 95% CI 0.98–1.68, p = 0.10), Orange (HR = 1.02, 95% CI 0.71–1.35, p = 0.91), Pink 0.97, 95% CI 0.64–1.30, p = 0.87) and Yellow (HR = 1.19, 95% CI 0.86–1.52, p = 0.30) (Fig. 6). With a hazard ratio of 1.67 (95% CI 1.51–1.83, p < 0.001), male mosquitoes were found to be 67% more likely to die than females each day. Mosquitoes that emerged on the second night of marking also had increased daily mortality risk (HR = 1.52, 95% CI 1.32–1.72, p < 0.001).

### Marking success of laboratory *Anopheles arabiensis*

On average, 85.9% (CI 82.5–87.9%) of *An. arabiensis* emerging in the laboratory experiments were marked. Yellow pigment was successfully transferred to 98.3% (CI 93.3–99.7%) of mosquitoes passing through the marking grid compared to 88.6% (CI 80.5–93.7%) with green pigment, 84.8% (CI 78.5–89.6%) with orange pigment, 82.8% (CI 75.6–88.2%) with blue pigment and 75% (CI 66.4–82.0%) with pink pigment. Tukey contrast indicated the only significant differences in the marking success were between yellow and three of the other four colours; pink (p < 0.001), orange (p = 0.011), and blue (p = 0.004). Mosquitoes emerging on the second day had a higher overall marking success with 87.9% (CI 84.0–91.0%) marked compared with 82.4% (CI 77.5–86.4%) on the first night (p = 0.011). The mosquitoes emerging on the second day may have spent longer under the marking unit and spent the daylight hours resting on the impregnated cloth, which could explain the higher marking rate. There was no difference in the marking rate of male (84.8%, CI 80.1–88.6%) and female mosquitoes (85.8%, CI 81.8–89.1%) (p = 0.52).

The number of mosquitoes marked during the MMRR field trial was calculated using a correction factor based on the average marking success in this semi-field experiment. While an individual correction factor could be applied for each colour, it was decided that the average of all colours (85.9%) was to be used as only the yellow pigment showed differences in marking success in select comparisons.

### Pigment transfer experiments

During the controlled pigment transfer experiment, transfer of pigment was not observed when collecting mosquitoes with CDC light traps (0/25 trials), Prokopacks (0/25 trials) or when aspirating mosquitoes individually (0/25 trials). However, pigment transfer was observed in 2/25 trials (8%) when aspirating mosquitoes in groups. In both of the two trials where pigment transfer was observed only one additional mosquito contained colour pigments. It was, therefore, concluded that pigment transfer through mosquito handling was unlikely to bias the results and no correction was needed.

### Natural breeding sites vs. pupae collection

The basic units over natural breeding sites marked an average of 0.6 *An. gambiae* s.l. per trap per day, with 15/15 mosquitoes being marked. The marking units that contained collected pupae marked an average of 4.4 *An. gambiae* s.l. per day, with 110/110 being marked. The latter method was, therefore, taken forward to the MMRR trial.

### MMRR field trial

#### Trapping

A total of 5116 mosquitoes were caught and identified during the 12 days of trapping. Table 1 summarizes the data by trap type, mosquito family and sex. Of the 770 *Anopheles* mosquitoes captured, 8 were morphologically identified as *An. funestus* s.l. and the remaining were *An. gambiae* s.l. Figure 5 shows Anopheline numbers by trap.

**Table 1:** Summary of the trapping results during the MMRR study

	Anopheline			
	CDC-LT		RBu	
	Male	Female	Male	Female
Total	7	632	60	71
Total/trap	0.32	31.60	2.00	2.37
Total/trap/night	0.02	1.66	0.11	0.12

#### Marking and recapture

502 mosquitoes emerged from the marking unit over five marking days (Table 2). A correction factor of 0.86, based on the average marking success in the semi-field experiment, was used to predict the number of marked mosquitoes. Of the 432 *An. gambiae* s.l. predicted to be marked, 41 *An. gambiae* s.l. were recaptured giving an overall recapture rate of 9.5%. If a 50:50 sex ratio is assumed, then the recapture rate for females was 16.7% but only 1.9% for males. There were variations between the colour cohorts ranging from a 4.1% recapture success with the orange cohort to 30.9% recapture success with the green cohort.

**Table 2:** Summary of the marking and recapture data

Dates	Pupae emerged and approximate number of mosquitoes marked					Number and proportion of marked recaptured mosquitoes					
	Colour	Number emerged	Number marked^a^	Male^b^	Female	Total	%	Female	%	Male	%
29/03/17	Pink	30	26	13	13	4	15.38	4	30.77	0	0
30/03/17	Blue	165	142	71	71	9	6.34	7	9.86	2	2.82
31/03/17	Orange	200	172	86	86	7	4.07	4	4.65	2	2.33
01/04/17	Green	64	55	27.5	27.5	17	30.91	17	61.82	0	0
02/04/17	Yellow	43	37	18.5	18.5	4	10.81	4	21.62	0	0
	Totals	502	432	216	216	41	9.49	36	16.67	4	1.85

The number of mosquitoes emerged through the unit was calculated from the number of pupae placed underneath the marking device and removing the number that remained the following day

^a^To calculate the number marked, a correction factor of 0.86 was applied to account for the marking success of the unit as observed in previous semi-field studies

^b^A 50:50 sex ratio of pupae is assumed to estimate the number of mosquitoes of each sex marked. An overall marking rate is given as well as data for each colour pigment for both sexes

#### Daily survival probability

**Fig. 7:**
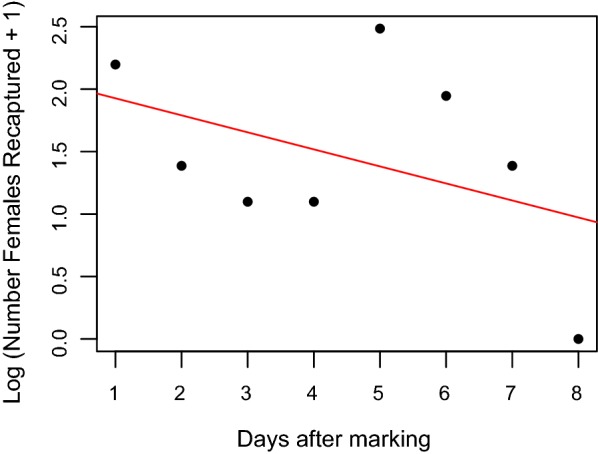
Daily survival of female *An. gambiae* s.l. in Yombo, Tanzania. Log transformed number of recaptured female *An. gambiae* s.l. by days after marking. The fitted exponential model predicting a daily survival probability of 0.87

Data from each colour cohort was combined to estimate the daily survival probability of the local female *An. gambiae* s.l. population. As there where eight recapture days following the final marking day survival rates were calculated for days 1–8 after marking. Insufficient data were available to measure male survival. The exponential model was fitted to the log_10_ (x + 1) number of marked mosquitoes recaptured against the days after marking (Fig. 7). The daily survival probability of female *An. gambiae* s.l. was 0.87 (95% CI 0.69–1.10). This equates to a life expectancy of 7.2 days.

#### Mean distance travelled

**Fig. 8:**
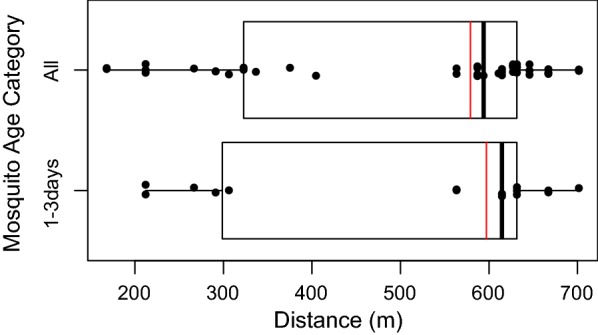
Boxplots visualizing the distribution of recaptured mosquitoes by distance. The red line indicates the MDT which includes a correction for sampling effort over distance. All mosquitoes captured (n = 41) are represented in the top boxplot with only mosquitoes ages 3-days or less (n = 15) represented in the bottom boxplot. Dots indicate individual mosquitoes

The distribution of recaptured mosquitoes by distance is shown in Fig. 8. The MDT, which accounts for sampling effort by distance was 579 m (95% CI 521–636 m) for female *An. gambiae* s.l. of any age. Using only female *An. gambiae* s.l. recaptured up to 3 days after marking, the “first flight” MDT travelled was 597 m (95% CI 509–685 m). Insufficient data were available to measure male MDT, however, the maximum male flight distance observed was 645 m.

## Discussion

This study demonstrates the successful use of a self-marking device in an MMRR study with African malaria vectors. The self-marking method described here has the demonstrated potential use as means to measure the dispersal and survival of wild African malaria vectors. Studies of this kind will provide much needed parameter estimates for malaria transmission models [42] and allow the assessment of novel control tools. While many other methods for MMRR studies are available, the method evaluated minimizes the effect of human interference on the survival and dispersal of wild *Anopheles* mosquitoes.

### Pigment load and survival

In the current study, a negative effect of the pigment on the survival of laboratory-reared anopheles mosquitoes was not observed which is in agreement with Niebylski and Meek’s observation when using a self-marking device for *Culex* mosquitoes [27]. They attributed this to the fact the self-marking unit results in a relatively low pigment load on the mosquito (5–15 pigment particles) and mainly on the legs, abdomen and thorax area—similar pigment loads on were observed on the laboratory-reared and wild *Anopheles* recaptured in this study. This is in contrast to traditional dusting methods that apply a large pigment load, often covering wings and the sensory organs on the head, potentially impacting survival [7]. Fluorescent pigments could also impact a mosquito’s host-seeking response and while the current evidence suggest the behaviour of *An. gambiae* s.s. is not affected by marking powders [24], further studies in this area are needed. The drawback to the lighter pigment load is that it is less obvious to the naked eye and it is more difficult to distinguish between the different pigment colours. UV torches and microscopes are, therefore, essential in identifying marked mosquitoes, which increases costs, time and workload for identification.

### Benefits of the self-marking unit

A self-marking unit has clear benefits of reducing the man-power involved in marking and eliminates any human-handling that may be detrimental to mosquito survival or natural behaviour. Ethically, it is a preferable method because there are no additional mosquitoes being added to the population and no additional genetic material. By marking field caught mosquitoes as they emerge from pupae with the current device it is possible to know the exact age of a marked mosquito when it is recovered, providing information that is rarely known in MMRR studies with wild mosquitoes.

Release methods may have important implications for MMRR studies but are often overlooked. It has been suggested that some mosquitoes memorize their home range and establish flight paths [17, 43] and, therefore, using laboratory-reared mosquitoes or releasing adult caught mosquitoes in areas away from their origin, may misrepresent true dispersal. Allowing mosquitoes to emerge close to their breeding site and disperse in their own time removes this stress factor and any arbitrary effects of release point and time on their behaviour.

### Study limitations

In the semi-field experiments around 14% of mosquitoes emerging from the device did not pick up the fluorescent pigment. It was possible to correct for this during analysis, however, complete marking success would be preferred. Niebylski and Meek [27] observed 100% marking success with their device and this may be due to the cloth used: they impregnated cheesecloth with pigment whereas in the current study, a more closely woven white cotton fabric was used as it was available locally. Modifications to the device could be made using materials to increase pigment transfer, for example, electrostatic gauze has been previously shown to mark mosquitoes with pigment after brief contact [44].

During the semi-field experiments, pigment transfer between mosquitoes was observed when manually aspirating them in groups. Due to a small diameter of the aspirator, mosquitoes occasionally get bunched together which may have caused the transfer of pigment. CDC-LT or Prokopack aspirators have much larger trapping containers and so mosquitoes are less likely to come into direct contact with each other and for pigment transfer to occur. Previous studies have observed low levels of pigment transfer when dusted mosquitoes are held together [7], but transfer has not been observed between mating pairs [7, 27]. Here it was observed that recapture methods can also cause pigment transfer and so should be considered when selecting recapture methods in a MMRR trial. During the trapping phase of the MMRR study mosquitoes carrying the same colour pigment was observed in the same trap on the same night on four occasions. This indicates the mosquitoes emerging on the same night arrived at the same house independently, however, contamination cannot be ruled out. In the semi-field experiments a small number of control mosquitoes were identified to be carrying colour pigment which could be due to contamination through forceps or microscope—it is, therefore, important to keep equipment scrupulously clean during mark-recapture experiments. It was also observed that swapping the colour grids each day was quite messy and a small amount of pigment from the previous day remained on the marking frame and surrounding area. This could be overcome by making more marking frames to keep the colours independent. On the rare occasion were two colour pigments on the same mosquito were observed, it was assumed that the mosquito emerged on the day of the most recent colour.

It was previously noted that a limitation of the self-marking device over a natural breeding site was that the number of mosquitoes emerging and the time of emergence could not be determined [45]. A possible solution would be to use an infrared counting system like the Biogents BG-Counter trap (Biogents AG, Regensburg, Germany) to count mosquitoes exiting the marking unit. However, to gain accurate estimates of the numbers emerging from the device, it is also possible to place pupae collected from several closely located breeding sites underneath the marking device and count pupal emergence as was done in this study and in a separate study with *Aedes albopictus,* [41]. Preliminary evaluations of the units indicated that it was impractical to mark the vectors of interest emerging from their natural breeding site. The basic marking units covered a breeding site of 0.25 m^2^ and marked 0.6 mosquitoes per day and so, assuming an equal emergence rate across the breeding site, an area roughly 20.8 m^2^ would need to be covered by emergence markers (84 self-marking units) to mark a minimum of 50 mosquitoes a day. This was not feasible in the breeding site under investigation and is unlikely for other malaria vector breeding sites were emergence rates might be even lower. The weekly emergence rate of anopheline mosquitoes in The Gambia has been estimated as 0.56 mosquitoes per m^2^ per week [46] and a study in the western Kenyan highlands estimated the emergence of *An. gambiae* as 1.82 per m^2^ per week [47]. In the current study, collecting pupae and stage four larvae from the surrounding area was very successful; however, the methodology is still dependent on there being relatively productive breeding sites available. Emergence rates from breeding sites will vary due to host of environmental factors and will vary dramatically between mosquito species. Before considering the use of the marking unit it is recommended that an entomological survey be conducted to determine if mosquitoes will emerge in sufficient numbers to be marked.

While this is certainly a limitation in areas with low mosquito numbers, overall the self-marking system provides a useful non-invasive alternative to traditional MMRR for measuring wild mosquito bionomics. The marking unit has also now been used in a MMRR study in Switzerland with *Aedes albopictus* [48].

### Mosquito survival estimates

Due to the small sampling area, clustering of houses in the MMRR study and short collection period, the distance and survival estimates calculated have to be interpreted with caution. MDT estimates are highly correlated to sampling area in MMRR studies [8] and survival estimates are influenced by mosquitoes leaving the study area. Despite this, the estimate of 0.87 as the daily survival of probability of female *An. gambiae* s.l. was similar to that found in previous studies. Gillies estimated the daily survival of *An. gambiae* to be 0.841 in an area of Tanzania slightly further inland. Other studies predicted daily survival to be 0.80–0.88 in Burkina Faso [18], 0.80 in Mali [11] and estimates ranging from 0.78 in another Tanzanian study [10] up to 0.95 in coastal Kenya [12]. The few estimates for *An. funestus* are more widely dispersed and range from a daily survival rate of 0.63 [10] to 0.837 [43] and up to 0.96 [12].

### Mosquito dispersal studies

Of the studies that previously measure dispersal in *An. gambiae* s.l., Costantini et al. sampled an area of similar size to the current study and estimated the daily dispersal of female *An. gambiae* s.l. to be 350–650 m which is line with 579 m overall MDT and 597 m “first flight” MDT measured here. Gillies on the other hand sampled up to 3.62 km away from the release point and estimated the mean dispersal distance (unadjusted) of female mosquitoes to be 1.02 and 1.58 km depending on their release point, in the centre or periphery of a village respectively. These distances were calculated over 23 days and, therefore, could include back and forth flight. To account for this, Gillies also looked at the dispersal after 1 day and found it to be 720 m in the central area which is similar to both the estimates of Costantini et al. [18] and those observed in the current study. While the calculation of MDT corrects for sampling effort over distance it assumes all locations are equally attractive to mosquitoes. Anthropophilic mosquitoes are attracted to areas of high population density [9, 49], and mosquito traps are necessarily differentially located in or around houses. The implications of this for estimation of MDT would merit further theoretical investigation. Gillies also suggests mosquito dispersal is highly influenced by the local topographical factors and therefore cautions against making generalizations outside a particular study setting. To gain a clearer understanding of mosquito dispersal there is a need for future MMRR studies to determine the factors influencing mosquito dispersal rather than just describing dispersal distances. As the current study was a proof of concept it was restricted in size; however, the self-marking unit has since been used in a large scale MMRR study (Saddler et al. in preparation) that will further add to the mosquito biology knowledge base.

## Conclusions

Despite the importance of mosquitoes for the transmission of malaria, there are relatively few empirical studies investigating key entomological parameters in wild mosquitoes. MMRR studies still have an important role in obtaining field estimates; and although there are a variety of methods available to mark mosquitoes, the self-marking unit described here has several logistical, ethical and biological benefits. The unit successfully marked wild *An. gambiae* s.l. males and females in sufficiently large numbers to justify its use in MMRR studies. The estimated daily survival probability of *An. gambiae* s.l. was 0.87 and mean dispersal distance was 597 m. These benefits may encourage further MMRR studies, allowing more accurate modelling and localized predictions of malaria transmission. In addition, the technique is simple enough to be used in vector control studies where population age and dispersal are important, including testing new vector control tools such as spatial repellents, gene drive mosquitoes and ATSB.

## Supplementary information

**Additional file 1.** MMRR field trial dataset. Contains data on individual recaptured mosquitoes, pupae numbers and mosquito trapping totals (marked and unmarked).

## Data Availability

Data generated and analysed during this study are included in this published article and its Additional file 1.

## Abbreviations

ALE: average life expectancy
ATSBs: attractive targeted sugar baits
CDC-LT: Centers for Disease Control and Prevention light trap
CF: correction factor
EAT: East African Time
ER: estimated recapture
GLMM: Generalized Linear Mixed Effects Models
IHI: Ifakara Health Institute
MDT: mean distance travelled
MMRR: mosquito mark-release-recapture
RBu: resting buckets
